# Examination of Duct Physiology in the Human Mammary Gland

**DOI:** 10.1371/journal.pone.0150653

**Published:** 2016-04-13

**Authors:** Dixie Mills, Ameer Gomberawalla, Eva J. Gordon, Julie Tondre, Mitra Nejad, Tinh Nguyen, Janice M. Pogoda, Jianyu Rao, Robert Chatterton, Susanne Henning, Susan M. Love

**Affiliations:** 1 Dr. Susan Love Research Foundation, 2811 Wilshire Blvd., Suite 500, Santa Monica, CA, 90403, United States of America; 2 Department of Preventive Medicine, Keck School of Medicine of the University of Southern California, Los Angeles, CA, 90089, United States of America; 3 Department of Pathology and Laboratory Medicine, David Geffen School of Medicine at the University of California Los Angeles, Los Angeles, CA, 90095, United States of America; 4 Department of Obstetrics and Gynecology, Northwestern University Feinberg School of Medicine, 710 North Fairbanks Court, Chicago, IL, 60611, United States of America; 5 Center for Human Nutrition, David Geffen School of Medicine at the University of California Los Angeles, Los Angeles, CA, 90095, United States of America; 6 Columbia University Medical Center, Department of Breast Surgery, New York, NY, United States of America; University of Torino, ITALY

## Abstract

**Background:**

The human breast comprise several ductal systems, or lobes, which contain a small amount of fluid containing cells, hormones, proteins and metabolites. The complex physiology of these ducts is likely a contributing factor to the development of breast cancer, especially given that the vast majority of breast cancers begin in a single lobular unit.

**Methods:**

We examined the levels of total protein, progesterone, estradiol, estrone sulfate, dehydroepiandrosterone sulfate, and macrophages in ductal fluid samples obtained from 3 ducts each in 78 women, sampled twice over a 6 month period. Samples were processed for both cytological and molecular analysis. Intraclass correlation coefficients and mixed models were utilized to identify significant data.

**Results:**

We found that the levels of these ductal fluid components were generally uncorrelated among ducts within a single breast and over time, suggesting that each lobe within the breast has a distinct physiology. However, we also found that estradiol was more correlated in women who were nulliparous or produced nipple aspirate fluid.

**Conclusions:**

Our results provide evidence that the microenvironment of any given lobular unit is unique to that individual unit, findings that may provide clues about the initiation and development of ductal carcinomas.

## Introduction

The human breast contains several ductal systems, or lobes, which serve as conduits for breast milk during lactation and are also the site of origin of the vast majority of breast cancers. Knowledge of the anatomy and physiology of the ductal systems is fundamental for issues surrounding normal mammary gland development and function as well as the diagnosis, treatment, and prevention of breast cancer and other pathological breast conditions. Despite these fundamental roles in lactation and disease, surprisingly little is known about human mammary duct anatomy and physiology, especially in the non-lactating breast where most breast cancers occur.

The majority of studies of human mammary duct anatomy have demonstrated that each ductal system is comprised of ducts that open at the nipple through one of 5–9 orifices [1–6] and branch into smaller and smaller passageways that ultimately terminate in lobules, where milk is made during lactation. Investigations into the origins and development of breast cancer in rodents have revealed that most breast cancers begin in the terminal ductal lobular units of an individual duct, which consist of a small segment of duct and a cluster of lobules [7]. However, the number of ductal systems varies in any given human breast, as do basic features within a particular ductal system such as morphology, size, and extent of branching [5, 8]. In addition, other fundamental properties of the breast, such as whether the lobes are monoclonal or polyclonal [9–11], or whether each lobe within a single breast contains the same composition and concentration of proteins, hormones, and other biomolecules, are strikingly underexplored. These properties could clearly affect the susceptibility of a specific lobe to cancer, underscoring the notion that a better understanding of the anatomy and physiology of the human breast could provide clues about cancer development.

Further evidence of the importance of focusing on the lobular unit rather than the breast is that non-invasive disease such as ductal carcinoma in situ (DCIS) is usually localized to a single ductal system [12]. Specific genetic and/or physiological factors within each lobe could predispose or promote malignant transformation, while analogous factors in a neighbouring lobe may support normal, healthy behaviour. To this point, the “theory of the sick lobe” posits that DCIS and invasive breast cancer are diseases of the lobe in which genetic factors predispose the duct to cancer, and environmental factors promote the development of the disease [13]. Moreover, Goldstein *et al*. have demonstrated that patients with high numbers of partially transformed columnar cell lesions, collectively termed monomorphic epithelial proliferations, near regions where cancerous tissue has been excised are more likely to have recurrences [14]. This suggests that individual lobes may have large areas of premalignant cells that can ultimately develop into cancer even after cancerous portions of the lobe have been removed.

The breast lobes contain a small amount of fluid that have numerous components, including cellular constituents such as ductal epithelial cells, macrophages, and foam cells; serum proteins such as albumin and immunoglobulins; hormones such as estrogens, androgens, progesterone, dehydroepiandrosterone sulfate (DHEAS), and prolactin; growth factors such as epidermal growth factor,transforming growth factor,and other biomolecules such as lipids, cholesterol and lactose [15]. The role of hormones in breast cancer development has prompted additional studies of hormone levels in ductal fluid [16–18], but the significance of the observed varying hormone concentrations and their correlations with other components of the ductal fluid is still poorly defined. Differing concentrations of these components could have profound effects on the local environment in the breast, influencing whether the ductal cells maintain a healthy disposition or progress toward a malignant phenotype. To this point, a key question is whether the relative amounts of these components vary among lobes within a given woman and vary over time within a single lobe.

Two methods commonly used to access ductal fluid from the mammary ducts are nipple aspiration and ductal lavage. Nipple aspiration involves massaging the breast, squeezing the nipple and applying gentle suction through an aspirator to elicit fluid from the ductal orifices. Whether or not a woman will express nipple aspirate fluid (NAF) depends on the woman as well as the experience and methods used by the clinician collecting the fluid. NAF typically emerges from just one or a few of the 5–9 ductal orifices on the nipple, and the properties of NAF, such as color and viscosity, differ from lobe to lobe, suggesting physiological differences among them. Because NAF or ductal fluid from a given subject is often pooled in studies, individual characteristics of fluid from different ducts of the same breast or woman have not been well characterized. An alternate method for sampling ductal fluid, called ductal lavage, enables minimally invasive sampling of the content of individual ducts [19, 20]. The technique involves local anesthetization of the nipple followed by duct dilation and cannulation. Saline (or another biocompatible fluid) is instilled into the ductal system through the nipple and subsequently recovered, bringing with it ductal epithelial cells and other components of the ductal fluid. This technique enables repeat sampling of the interior of a single lobe and is thus an appealing approach for investigating the physiology of individual lobes.

In this study, our primary goal was to evaluate the correlation between samples of ductal fluid from different lobes within a single breast and within a given lobe sampled twice over 6 months using ductal lavage. We measured ductal fluid levels of total protein, macrophages, and the hormones estrone sulfate, dehydroepiandrosterone sulfate (DHEAS), progesterone, and estradiol. We assessed within-subject correlations among protein, macrophage, and hormone levels and examined whether these ductal fluid components might be affected by individual characteristics such as age, parity, menopausal status, and production of NAF. Our findings have implications in understanding the physiology of the normal human mammary gland as well as the etiology of breast diseases including cancer.

## Materials and Methods

### Subject recruitment

Seventy-eight women from a community setting with current non-suspicious mammograms and unselected for risk were recruited to this study approved by the ethics committee and IRB Independent Review Consulting, Inc. (IRC study # 05142). Recruitment took place through local speaking engagements, news articles, and word of mouth. Exclusion criteria included current pregnancy, lactation, chemotherapy, subareolar surgery, hormone use, and active breast infection. The study design was explained and informed consent was obtained from all subjects prior to their participation.

### Sample collection

With a small number of noted exceptions, ductal lavage was performed by Dr. Susan Love for the first 15 subjects and by Dr. Dixie Mills for the remaining 63 subjects. Prior to ductal lavage, expression of NAF was attempted to identify which, if any, ducts were fluid-yielding, and this information was recorded in the subject’s chart. Menopausal status was self-reported either by lack of periods for 1 year or surgically. Additionally, no subject’s menopausal status changed during the course of this study.

For the ductal lavage procedure, subjects received a 1 cubic centimeter (cc) injection of lidocaine buffered with sodium bicarbonate (9:1 ratio) directly into the nipple using a tuberculin syringe with a 30-gauge needle. Additional anesthetic was given throughout the procedure if the subject regained sensitivity, and intranipple Marcaine (approximately 1 cc) was injected at the end of the procedure to prolong the anesthetic effect. After the nipple was engorged with anesthetic, ductal orifices were more readily identifiable by dimpling of the skin or the presence of fluid droplets. Upward traction on the nipple straightened the subareolar ducts increasing the ease of cannulation.

Ductoscopy was performed in the standard manner with a 0.9-mm diameter, 10-cm long microendoscope (Acueity, Hayward, CA) to document a ductal lumen or perforation. Regardless of whether a duct was observed to be intact or perforated, the ductoscope was removed and replaced with a microcatheter (Cytyc, Marlborough, MA) that had been flushed with lidocaine and standard ductal lavage was performed under ultrasound guidance. Lavage was performed on 2 to 4 lobes in one breast under real-time monitoring with high-definition ultrasound (HDI Philips 5000 SonoCt with Real-time Compound Imaging) equipped with a 5–12 MHz linear array transducer. The double-lumen catheter tip was located and visualized on the monitor to confirm its presence in an intact duct, and a total of 10 to 20 cc of saline was slowly instilled. Approximately 5 to 10 cc of ductal fluid was retrieved for subsequent analysis.

A nipple grid was used to document the location of the lavaged ducts and a photo was taken of a knotted suture in the ducts that had been lavaged. At a follow-up procedure 6 months later, the same lobes were lavaged by the same surgeon who performed the initial procedure. This allowed analysis of within-duct variability while minimizing potential variation due to clinician performing the procedure. Lavaged lobes included both those that produced NAF (wet lobes) and those that did not produce NAF (dry lobes).

### Sample analysis

Ductal lavage samples were processed for both cytological and molecular analysis. Immediately after collection, lavage effluent was centrifuged at 2500 rpm for 10 minutes to separate cells from fluid components. Ductal lavage supernatant from each lobe was analyzed for total protein and hormone levels. Samples were analyzed at the University of California, Los Angeles (UCLA) in the laboratory of Dr. Susanne Henning. Macrophage cell number for all samples were analyzed in the laboratory of Dr. Jianyu Rao on Papanicolaou-stained slides prepared from the cell pellet.

Chromatography mass spectrometry (GC-MS/MS) was used to analyze estradiol and progesterone in the ductal lavage samples. 17β-estradiol (Sigma, E-2758, St. Louis MO) and progesterone (E9145 Sigma), dissolved in methanol, was used to establish a standard curve. Deuterated estradiol (17β-estradiol-d3, Sigma #491187) and progesterone-d9 (CDN Isotope Inc. D-5385, Quebec Canada) were added as internal standard to 2–4.5 mL of ductal lavage fluid samples prior to column concentration. Samples were extracted on a Supelco Discovery solid phase extraction DPA-6S column (Supelco, Bellefonte, PA), pre-conditioned with ethyl acetate, methanol, water and plasmalyte and eluted with ethyl acetate/methanol (4:1). 50 μL of eluate was used for the progesterone analysis and directly injected into the GC/MS. The remaining eluate was dried under nitrogen stream and redissolved in N,O-bis(trimethylsilyl)trifluoroacetamide (BSTFA) with 1% trimethyl- chlorosilane (TMCS) catalyst (Supelco, Bellefonte PA,#3–3148) for derivatization at 65°C for 30 min. The derivatized samples were dried under nitrogen stream and reconstituted with 50μL hexane. Analyses were carried out using a ThermoQuest TRACETM 2000 gas chromatography coupled with a ThermoQuest TRACETM MS. Sample injection (1 μL) was in splitless mode. A Restek RTx-5 column (15 m×0.25 μm×250 μm) GC column was used. Helium carrier gas was maintained at a constant flow rate of 1.0 mL/min. The GC column temperature was programmed from 60 (initial equilibrium time 2 min) to 280°C via a ramp of 10°C/min, 280–320°C via a ramp of 40°C/min and maintained at 320°C for 3 min. The MS detection was by electron impact ionisation and operated in full scan mode for qualitative analysis or selected ion monitoring (SIM) mode for quantitative analysis. The limit of sensitivity was 8.8 pg/mL of progesterone and 0.1 pg/mL for estradiol.

Estrone sulfate in 100 μL of lavage fluid was analysed using the radioimmunoassay from Diagnostic Systems Laboratories (now Beckman Coulter) (Fullerton, CA) DSL5400 according to manufacturer’s instructions. The DSL estrone sulfate radioimmunoassay uses a rabbit antibody preparation with high specificity for estrone sulfate. The procedure follows the basic principle of radioimmunoassay where there is competition between a radioactive and a non-radioactive antigen for a fixed number of antibody binding sites. The amount of [^125^I]-labeled estrone sulfate bound to the antibody is inversely proportional to the concentration of unlabeled estrone sulfate present. The separation of the free and bound antigen is achieved by using a double antibody system. The intra-assay precision was 4.7, 4.6 and 9.2% for concentrations of 59.3, 9.0 and 0.35 ng/mL, respectively, and inter-assay precision was 5.5, 5.1 and 8.8 for concentrations of 11.3, 0.5 and 0.08 ng/mL of estronesulfate, respectively. The limit of sensitivity was 0.456 pg/mL.

DHEAS in 10 μL of DLF was analyzed using the radioimmunoassay from Diagnostic Systems Laboratories (now Beckman Coulter) (Fullerton, CA) DSL3500. The intra-assay precision was 6.3, 7.8 and 9.4% for concentrations of 37.4, 14.5 and 1.9 μg/dL of DHEAS, respectively, and inter-assay precision was 9.9, 10 and 9.6 for concentrations of 55.5, 17.3 and 2.0 μg/dL of DHEAS, respectively. The limit of sensitivity was 88 pg/mL.

Protein concentration was determined using the BioRad Protein Assay Dye Concentrate (500–0006) and protein standard (BioRad 500–0007) according to the standard protocol.

For cytological analysis, the cells were resuspended in 20 mL of Preservcyt solution (Cytyc, Marlborough, MA). Samples were submitted in a blinded fashion to a central laboratory and were analyzed for macrophage on Papanicolaou-stained slides.

### Statistical analysis

Given the relatively large proportion of analyte values below the detection limit (BDL, up to half, depending on the analyte) a multiple imputation technique was used for analysis [21]. Specifically, ten data sets were created using clustered bootstrap sampling of the log-transformed original data [22]. The maximum likelihood estimate of the mean of each analyte, by visit, was calculated using spreadsheet software [23], and this was then used to derive the mean of the truncated portion of the distribution below the BDL [24]. Under the assumption that within-subject and within-duct correlation was the same for BDL values as for non-BDL values, analyte-specific BDL observations were imputed as follows: (1) using only non-BDL data, the procedure MIXED in SAS v9.2 (SAS Institute, Inc., Cary, NC) was used in an unconditional means model with “subject” and “duct” as random effects, (2) within-subject and within-duct correlations were taken from the correlation matrix of the resulting estimated random effects covariance (“V”) matrix (SAS/STAT v9.2 User’s Manual) [25], (3) a vector of 6 correlated observations (corresponding to observations from 3 ducts over 2 visits) was randomly generated using the mean of the truncated distribution and the elements of the Cholesky decomposition of the V correlation matrix, (4) depending on the vector position of the BDL observation relative to duct (first, second, or third duct within the given subject) and visit (first or second), an imputed value was selected from the randomly generated vector to replace the BDL observation. All analyses involving analytes were based on combined estimates from the ten bootstrapped data sets [26].

As a sensitivity analysis, BDL observations were also handled using three alternative methods: (1) excluding BDL observations from analysis (i.e., no assumptions about BDL correlation structure but may induce bias due to the exclusion of data), (2) imputing BDL observations with random draws from a univariate distribution of values from 0 to the BDL value (i.e., assumes no correlation within BDL observations or between BDL and non-BDL observations), and (3) imputing BDL observations with the midpoint value between 0 and the BDL value (i.e., assumes perfect correlation within BDL observations but no correlation between BDL and non-BDL observations).

Within-lobe and within-subject correlations in observed analyte levels were evaluated by calculating intraclass correlation coefficients (ICCs); specifically, within-lobe and within-subject ICCs were calculated as the ratio of the random effects covariance parameter estimate for “lobe” and “subject,” respectively, to the sum of “lobe,” “subject,” and residual covariance parameter estimates. Thus, the ICC represents the proportion of overall variability in analyte levels attributable to the variability in analyte levels between lobes or subjects. For example, a high within-subject ICC implies that a large amount of the variability observed in analyte levels is due the variability that exists between individual subjects rather than within subjects, which in turn implies that observations within subjects are highly correlated. An unstructured covariance matrix was specified. Ninety-five percent confidence intervals for ICCs were computed as the 2.5 and 97.5 percentiles of the ICC distribution resulting from 1000 trials of bootstrap-sampling from each of the 10 bootstrapped data sets and computing the ICC as described above.

Differences in analyte levels by subject- and duct-level factors were analyzed using mixed models. Relationships between pairs of analytes were evaluated by Pearson correlation coefficients. Covariance between analytes was not considered in the BDL imputation described above; thus, reported correlations between pairs of analytes are conservative.

NAF was analyzed as an indicator variable both at the duct and subject level. Duct-level NAF was defined as “yes” if the lobe produced NAF at either visit and as “no” otherwise. Subject-level NAF was defined as “yes” if any lobe at either visit produced NAF and as “no” otherwise. Differences in macrophage levels by lobe- and subject-level NAF were tested using the SAS procedure GENMOD with a binomial probability distribution and the logit link function. Generalized estimating equations solutions were requested by declaring “subject” as a repeated effect, with compound symmetry working correlation structures.

## Results

Ductal lavage was performed on 206 ducts in 78 women, with the number of lobes per woman ranging from 2 to 4. Most women (94%) contributed 3 ducts, and most ducts (79%) were sampled twice, six months apart, for a total sample size of 419; of these, data were available for 397 (95%) ductal samples. Study participant characteristics are shown in Table 1. Mean age among participants was 52 years; most had low breast cancer risk, were parous, and did not produce NAF, and 44% were post-menopausal. Lobar characteristics are shown in Table 2. Among lobes sampled from women who were not post-menopausal, there was a fairly even distribution between follicular and luteal cycle at the time of lavage. Most lobeshad fewer than 10 macrophages and did not produce NAF. About 8% were perforated. Percentage of BDL ductal samples by analyte are shown in Table 3.

**Table 1:** Study Participant Characteristics.

Parameter	All Participants (N = 78)	
Age (yrs)		
N	78	
Mean (SD)	51.7	(11.72)
Median	51.5	
Interquartile range	44.0	-59.0
Range	22	-76
Breast cancer risk^1^ [n (%)]		
Low	39	(50.0)
High	26	(33.3)
Breast cancer	9	(11.5)
Unknown	4	(5.1)
Nulliparous [n (%)]		
No	49	(62.8)
Yes	29	(37.2)
Menopause status [n (%)]		
Pre	44	(56.4)
Post	34	(43.6)
NAF Producer [n (%)]		
No	62	(79.5)
Yes	16	(20.5)

^1^Based on Gail Index (High = Gail 1.66 or higher).

**Table 2:** Duct Characteristics.

Parameter	Duct Samples (N = 397)	
Anatomy		
Duct	290	(73.4)
Perforation	30	(7.6)
Unknown	75	(19.0)
Cycle		
Follicular	79	(20.0)
Luteal	98	(24.8)
N/A	212	(53.7)
Unknown	6	(1.5)
NAF Producer		
No	362	(91.6)
Yes	33	(8.4)
Macrophages		
< 10	256	(64.8)
> = 10	48	(12.2)
Unknown	91	(23.0)

**Table 3:** Percentage of Ductal Samples Below the Detection Limit.

Analyte	Detection Limit	N	Number (%) Below the Detection Limit
Protein	0.1 μg/mL	397	51 (12.8)
Progesterone	8.8 pg/mL	394	137 (34.8)
Estradiol	0.1 pg/mL	394	205 (52.0)
Estrone sulfate	0.456 pg/mL	397	213 (53.7)
DHEAS	88 pg/mL	396	81 (20.5)

Ductal levels of total protein, estrone sulfate, DHEAS, and estradiol were similar between visits (data not shown) but progesterone levels were lower at baseline than they were 6 months later (mean (95% CI) log pg/mL progesterone = 1.97 (0.84, 3.09) at baseline, 3.01 (2.08, 3.94) after 6 months; p = 0.053). Analyte levels did not differ by the participant-level factors of breast cancer risk, parity, menopause status, or whether or not the participant produced NAF (data not shown); however, differences in estradiol levels by age were borderline significant, with younger women having higher estradiol levels than older women (mean (95% CI) log pg/mL estradiol = -1.37 (-2.71, -0.02) < 45 years old, -2.81 (-4.04, -1.58) > 55 years old; p = 0.07). Analyte levels did not significantly differ by the lobe-level factors of anatomy (duct vs. perforation), cycle (follicular vs. luteal), NAF production, or number of macrophages, with the following exceptions: lobes in the follicular phase had significantly lower protein levels than ducts in the luteal phase (mean (95% CI) log μg/mL protein = 0.99 (0.13, 1.84) follicular, 2.13 (1.38, 2.89) luteal; p = 0.03), and lobes with fewer macrophages had significantly lower protein levels than ducts with more macrophages (mean (95% CI) log μg/mL protein = 1.26 (0.80, 1.73) < 10 macrophages, 2.99 (2.11, 3.87) ≥ 10 macrophages; p = 0.002). Differences in estrone sulfate levels by cycle were borderline significant, with follicular ducts having lower estrone sulfate levels than luteal ducts (mean (95% CI) log pg/mL estrone sulfate = -1.88 (-3.38, -0.39) follicular, -0.53 (-1.82, 0.75) luteal; p = 0.08). Differences in progesterone levels by number of macrophages were borderline significant, with low-macrophage ducts having lower progesterone levels than high-macrophage ducts (mean (95% CI) log pg/mL progesterone = 3.02 (2.07, 3.97) low-macrophage, 4.48 (3.01, 5.96) high-macrophage; p = 0.06).

The largest degree of correlation, either positive or negative, between analytes was between progesterone and estradiol (r = 0.23). Within-subject correlations among protein, progesterone, estradiol, estrone sulfate, and DHEAS levels, both overall and stratified by participant-level characteristics, are shown in Table 4. Overall ICCs ranged from 0.09 (95% CI = 0.04, 0.18) for estrone sulfate to 0.24 (95% CI = 0.18, 0.35) for estradiol. The most notable ICC differences by participant-level characteristics were that estradiol ICC was higher in nulliparous compared to parous women and in NAF producers compared to non-NAF producers, and progesterone ICC was higher in NAF producers compared to non-NAF producers.

**Table 4:** Intraclass Correlation Coefficients (ICCs) and 95% Confidence Intervals (CIs) by Analyte and Participant Characteristics.^1^

Participant	Protein			Progesterone			Estradiol			Estrone Sulfate			DHEAS		
Characteristic	n	ICC	95% CI	n	ICC	95% CI	n	ICC	95% CI	n	ICC	95% CI	n	ICC	95% CI
Overall	397	0.19	0.11, 0.32	394	0.17	0.12, 0.31	394	0.24	0.18, 0.35	397	0.09	0.04, 0.18	396	0.20	0.13, 0.30
Breast cancer risk															
Higher	184	0.14	0.03, 0.25	183	0.11	0.04, 0.31	182	0.19	0.12, 0.36	184	0.04	0.00, 0.15	184	0.27	0.15, 0.41
Lower	190	0.23	0.08, 0.42	188	0.15	0.06, 0.32	190	0.29	0.16, 0.42	190	0.14	0.05, 0.27	189	0.15	0.03, 0.28
Nulliparous															
No	240	0.23	0.11, 0.37	239	0.14	0.10, 0.32	239	0.12	0.06, 0.26	240	0.09	0.02, 0.20	240	0.24	0.15, 0.36
Yes	157	0.12	0.01, 0.33	155	0.23	0.08, 0.40	155	0.40	0.26, 0.56	157	0.09	0.01, 0.25	156	0.14	0.00, 0.28
Menopause status															
Pre	211	0.16	0.03, 0.34	209	0.22	0.12, 0.38	210	0.29	0.18, 0.42	211	0.13	0.04, 0.25	210	0.14	0.06, 0.26
Post	186	0.23	0.12, 0.39	185	0.11	0.03, 0.31	184	0.15	0.09, 0.32	186	0.04	0.00, 0.16	186	0.26	0.15, 0.40
NAF producer															
No	322	0.19	0.10, 0.31	319	0.14	0.08, 0.28	319	0.19	0.12, 0.31	322	0.07	0.02, 0.18	322	0.22	0.15, 0.33
Yes	75	0.22	0.00, 0.53	75	0.39	0.20, 0.66	75	0.47	0.24, 0.70	75	0.16	0.01, 0.33	74	0.13	0.00, 0.31
Macrophage levels															
Low	244	0.10	0.00, 0.28	241	0.15	0.07, 0.31	241	0.27	0.16, 0.41	244	0.10	0.03, 0.24	243	0.21	0.13, 0.34
High	153	0.29	0.16, 0.42	153	0.17	0.07, 0.36	153	0.17	0.08, 0.32	153	0.08	0.00, 0.19	153	0.19	0.06, 0.34

^1^ICCs are means over 10 imputed data sets. 95% CIs are based on 2.5 and 97.5 percentiles of the ICC distribution over 1000 bootstrapped samples of the 10 imputed data sets.

Within-subject ICCs by analytical method for dealing with BDL observations are shown in Table 5. Predictably, ICC point estimates were most affected by method for the two analytes with the highest percentage of BDL observations, estradiol and estrone sulfate, but overall there was little effect of method on ICC point estimates.

**Table 5:** Intraclass Correlation Coefficients (ICCs) and 95% Confidence Intervals (CIs) by Analyte and Statistical Method to Account for Observations Below the Detection Limit (BDL).

	Protein (13% BDL)			Progesterone (35% BDL)			Estradiol(52% BDL)			Estrone Sulfate(54% BDL)			DHEAS(21% BDL)		
Method	n	ICC	95% CI	n	ICC	95% CI	n	ICC	95% CI	n	ICC	95% CI	n	ICC	95% CI
Multiple Imputation^1^	397	0.19	0.11, 0.32	394	0.17	0.12, 0.31	394	0.24	0.18, 0.35	397	0.09	0.04, 0.18	396	0.20	0.13, 0.30
Exclude^2^	346	0.21	0.05, 0.35	257	0.19	0.02, 0.42	189	0.29	0.11, 0.50	184	0.13	0.00, 0.39	315	0.22	0.08, 0.41
Random^3^	397	0.17	0.03, 0.37	394	0.17	0.04, 0.36	394	0.22	0.13, 0.41	397	0.07	0.00, 0.24	396	0.22	0.08, 0.36
Midpoint^4^	397	0.20	0.11, 0.30	394	0.17	0.11, 0.37	394	0.25	0.17, 0.42	397	0.09	0.03, 0.31	396	0.23	0.14, 0.33

^1^ICCs are means over 10 data sets with BDL values imputed. 95% CIs are based on 2.5 and 97.5 percentiles of the ICC distribution over 1000 bootstrapped samples of the 10 imputed data sets.

^2^BDL observations excluded.

^3^BDL observations imputed by random draw from a univariate distribution of values from 0 to BDL value.

^4^BDL observations imputed by midpoint value between 0 and BDL value.

For all analytes, correlation among analyte levels measured within the same lobe over two visits was virtually zero (data not shown).

Macrophage levels by subject- and lobe-level NAF are shown in Table 6. Lavage fluid from wet lobes had somewhat more macrophages than lavage fluid from dry lobes, though the difference was not significant (p = 0.67). Similarly, there was a non-significant greater number of macrophages in ductal lavage fluid from women who produced NAF compared to those who did not produce NAF (p = 0.33).

**Table 6:** Number of Macrophages by Duct- and Participant-Level NAF Status.

	Duct NAF = No		Duct NAF = Yes		Participant NAF = No		Participant NAF = Yes	
Number of	(N = 383)		(N = 36)		(N = 339)		(N = 80)	
Macrophages	n	(%)	n	(%)	n	(%)	n	(%)
< 10	237	(85.3)	19	(73.1)	212	(85.5)	44	(78.6)
10–50	18	(6.5)	2	(7.7)	14	(5.6)	6	(10.7)
50–100	7	(2.5)	2	(7.7)	7	(2.8)	2	(3.6)
100–1000	9	(3.2)	2	(7.7)	9	(3.6)	2	(3.6)
> 1000	7	(2.5)	1	(3.8)	6	(2.4)	2	(3.6)
Unknown	105		10		91		24	

Note: p-value for difference in number of macrophages = 0.67 by duct-level NAF and 0.33 by participant-level NAF.

## Discussion

Our examination of protein and certain steroid hormone levels in human mammary lobes suggests that these components are generally not highly correlated within subjects, although there may be differences in levels of certain hormones by factors that have been associated with breast cancer risk, such as parity and NAF production. This finding has several far-reaching ramifications for understanding the development and function of the normal mammary gland and for efforts to determine the etiology and progression of breast disease, including breast cancer. Lack of correlation among lobes intimates that the signaling and communication events orchestrated by components of the ductal cells and fluid may be different in each duct, which could result in individual duct differences in susceptibility to disease. These unique lobar characteristics also gives more evidence that the breast lobes are individually different and lack a significant number of anastomoses/connections. This theory is more in line with observations from imaging studies like galactography, injections, and ductoscopy, and contrary to studies that favour such anastomoses. Additionally, the fact that we observed that some hormones were more correlated within nulliparous and NAF producing women may suggest that these hormones may be the targets for future research on risk management, as these factors have previously been shown to be associated with increased breast cancer risk [27].

Other researchers have investigated within-subject correlation of biomarkers from ducts. Chatterton *et al*. observed higher levels of correlation than we did; in their study, the variability of estadiol, estrone sulfate, androstenedione, DHEA, DHEAS, and progesterone levels among ducts within breasts averaged 51% less than that between women [28]. Participants in this study were either breast cancer patients or were at high risk for breast cancer (GAIL > 1.6). Interestingly, in our study, the highest within-breast correlations, albeit limited to estradiol, were observed in nulliparous women and in women who produced NAF, factors that have been associated with higher risk of breast cancer.Estradiol ICCs in our study were 0.40 in nulliparous women and 0.47 in NAF-producing women, while Chatterton *et al*. observed an ICC of 0.33 for estradiol in their high-risk study population (the lowest ICC they observed among all analytes).

Bhandare *et al*. also observed relatively high within-subject correlation for estradiol as well as androstenedione, again in high-risk women [17]. However, they also determined that associations between certain biomarkers were stronger when the analyses were done within individual ducts than when done within a single woman. For example, in ductal lavage fluid, levels of epidermal growth factor were significantly associated with estrone sulfate levels, and this association was more robust within a single duct than within an individual woman, suggesting some independence between ducts.

Both the Chatterton and Bhandare studies differed methodologically from our study. For example, in both of these studies, hormone levels were expressed per milligram of protein in the fluid. This approach was used because, with the lavage procedure, the actual volume of ductal fluid is not known because it is diluted by a large volume of buffer during the procedure [18, 27]; thus, expressing hormone levels relative to the amount of ductal fluid in the sample is not possible. They theorized that, because the protein concentration in colostrum is relatively constant, it is likely constant in lavage fluid as well, and in their analysis they demonstrated the validity of this approach based on the correlations in their samples between hormone levels expressed as per milliliter fluid and per milligram protein. This method assumes a constant ratio of hormone to protein across protein levels. In our data, this was not the case, as the ratio of hormone to protein decreased with increasing levels of protein. Because a relatively small amount of ductal fluid is diluted to a much larger and relatively consistent volume, we felt it was more appropriate to express hormone levels as milligrams per milliliter of lavage fluid. Also, rather than exclude BDL data in our study, we explored alternative statistical methods that allowed us to retain these data in our analyses. In both the Chatterton and Bhandere studies, samples with insufficient cells were excluded. These differences between our studies complicate meaningful comparisons of our results.

Chatterton *et al*. also measured changes in concentrations of various hormones and proteins in NAF and ductal lavage fluid over time and found that levels were relatively stable within individual lobes over 15 months, especially for estrone sulfate and DHEAS [18]. In contrast, we observed very little correlation in hormone levels within individual ducts over a 6 month period. Chatterton *et al*. also found that correlations were slightly higher within a single breast over time than between the left and right breast at a single time, suggesting that local factors may play a role in determining hormone and protein levels in NAF. However, since this study did not compare levels of substances between ducts within a single breast at a given time, and our study did not collect fluid from both breasts of the same woman, it is again difficult to make direct comparisons between our findings.

Patil *et al*. examined epithelial cells in ductal lavage fluid in 65 high-risk women twice over a six month period and found poor reproducibility of cell yield and cytology findings within the same duct [29, 30]. The authors hypothesized that the higher proportion of lower cell yield from the second visit suggests that ductal lavage may have stripped the epithelium and/or weakened the duct walls, compromising the integrity of the second sampling. In addition, they suggest that the poor cytological reproducibility observed indicates that ductal lavage may not be an optimal method for serial evaluation of the ductal epithelium. The fact that we did not observe decreased mean hormone and total protein levels over the 6-month period of our study suggests that if the lavage procedure does cause prolonged epithelial stripping, this does not lead to a systematic decline in hormone and protein levels in ductal fluid. We hypothesize that variability in the ductal fluid is due to an inherent dynamic and variable physiology in the ducts.

Our finding that women who produced NAF had somewhat higher numbers of macrophages in their ducts is intriguing given the links between inflammation, the immune system, and cancer [31, 32]. Though the physiology of NAF is not well understood, there is some evidence that women who produce NAF are at increased risk for breast cancer [33, 34]. The possible contribution of macrophages to this complex physiology is unclear, but our data provide another potential piece to the multifaceted puzzle of factors that contribute to breast cancer development.

There were limitations to our study. A relatively large proportion of data were BDL. While we used several different statistical approaches to incorporating these data into our analysis and observed consistent ICCs regardless of method, the correlation structure of these data are unknown and the assumptions required by these various statistical methods may not be biologically correct. As mentioned previously, it is not possible to measure the actual volume of ductal fluid from the lavage process, which complicates standardization. We chose to not standardize analyte levels and instead used actual measured amounts of total protein and each hormone in a given sample, which assumes that each sample contained the same amount of fluid. It is not possible to quantify the effect this may have had on our findings.

On the other hand, a strength of our study was the demonstration that majority of the ducts were able to be sampled twice without a drop in yield, demonstrating further data that follow up within the same duct is feasible. This finding also highlights the potential of an intraductal approach for evaluation of breast health [34–36]. Intraductal methods such as ductal lavage and ductoscopy offer a means to access, evaluate, and even treat individual mammary ducts. The local nature of non-invasive breast cancer and other breast conditions such as papillomas, coupled with our evidence that duct physiology may differ from duct to duct, espouses the idea that intraductal methods can be more effective for managing breast conditions than many of the invasive, surgical or systemic options in current use.

In summary, our study demonstrated that the hormonal and cellular environment of individual ducts within the same woman and within the same duct over time is variable. Individual ducts likely require individual attention, and further investigation of these distinct ducts could be the key in identifying the origins of breast tumorigenesis, giving a target for a potential cure.
